# Racial and Ethnic Disparities in NAFLD: Harnessing Epigenetic and Gut Microbiota Pathways for Targeted Therapeutic Approaches

**DOI:** 10.3390/biom15050669

**Published:** 2025-05-05

**Authors:** Mohamed Zaiou, Olivier Joubert

**Affiliations:** Université de Lorraine, CNRS, IJL, F-54000 Nancy, France; olivier.joubert@univ-lorraine.fr

**Keywords:** NAFLD, health disparities, epigenetics, DNA methylation, MASLD, dysbiosis

## Abstract

Nonalcoholic fatty liver disease (NAFLD) is a growing global health concern, impacting approximately 32.4% of the worldwide population. As a disease linked to metabolic dysfunction, NAFLD continues to rise alongside global increases in obesity, type 2 diabetes mellitus (T2DM), and metabolic syndrome. There is considerable evidence indicating that NAFLD disproportionately affects racial, ethnic, and minority groups, although the exact reasons for these disparities remain elusive. Contributing factors to this disease may include socioeconomic status, cultural influences, stress, genetic factors, and lifestyle choices. Emerging evidence suggests that these causal factors could influence epigenetic mechanisms, particularly DNA methylation and histone modifications, as well as the composition and diversity of gut microbiota. Nevertheless, there is a scarcity of research that comprehensively examines the interplay between epigenetic changes and gut microbiome variations in relation to NAFLD disparities across different racial and ethnic populations globally. This paper intends to (i) explore the connections between NAFLD, ethnic disparities, gut microbiota composition, and epigenetic alterations, while reviewing pertinent studies that illustrate how these factors contribute to health inequities among various ethnic groups impacted by this disease; (ii) explore potential therapeutic targets and biomarkers to advance the management of NAFLD; and (iii) provide insights to enhance our understanding of the mechanisms associated with this disease, thereby promoting further research in this field. Advancements in this area are anticipated to enhance our understanding of disease susceptibilities in at-risk groups and to provide new therapeutic options for NAFLD and its associated complications.

## 1. Introduction

Nonalcoholic fatty liver disease (NAFLD) is a prevalent chronic condition marked by an abnormal accumulation of fat in the liver, unrelated to alcohol intake. The incidence of NAFLD is increasing globally, now affecting over 30% of the population worldwide [1]. This condition encompasses a spectrum of liver disorders, ranging from simple steatosis (commonly referred to as fatty liver) to nonalcoholic steatohepatitis (NASH), which is characterized by liver inflammation, hepatocyte injury, and fibrosis [2]. The transition from simple hepatic steatosis to NASH, and ultimately to cirrhosis and hepatocellular carcinoma (HCC), is driven by various mechanisms, including fat deposition in the liver, insulin resistance, inflammation, and fibrosis [3]. NAFLD is now recognized as a hepatic manifestation of metabolic syndrome (MetS), which is associated with obesity, type 2 diabetes mellitus (T2DM), insulin resistance, and dyslipidemia [4], making it a significant global public health concern.

In 2020, international consensus guidelines recommended the reclassification of NAFLD to metabolic-associated fatty liver disease (MAFLD), which is based on specific diagnostic criteria [5]. In June 2023, a prominent panel of experts, supported by leading liver societies from Europe and the United States, proposed and adopted the nomenclature metabolic dysfunction-associated steatotic liver disease (MASLD) as a replacement for NAFLD [6]. This new terminology refers to the occurrence of hepatic steatosis alongside at least one cardiometabolic risk factor, such as overweight/obesity, T2DM, or indicators of metabolic dysregulation. Although the proposed definitions differ, they exhibit considerable overlap, as nearly 99% of individuals diagnosed with NAFLD would also be categorized as having MASLD [7,8]. Both terms encompass critical features of liver disease associated with cardiometabolic risk factors, which significantly influence both overall and liver-related mortality rates. In this context, recent research indicates that the newly adopted term MASLD largely retains the connotations associated with NAFLD [9]. To achieve thorough coverage of the literature in this review, we will continue to utilize the established terminology, NAFLD. We consider this approach essential as we seek to summarize all relevant findings related to ethnic disparities in NAFLD and that were published prior to the adoption of the new nomenclature.

The precise pathogenic mechanism of NAFLD remains unclear; however, several factors are believed to play a role, including genetic susceptibility, dietary habits, oxidative stress, de novo lipogenesis, proinflammatory cytokines, insulin resistance, the gut–liver axis and dysbiosis. Often, NAFLD is asymptomatic, especially in its early stages, which can result in patients being unaware of their condition until liver fat is identified through routine liver enzyme tests or imaging studies. This complex disease also has implications for extrahepatic conditions such as T2DM, cardiovascular disease, and chronic kidney disease, presenting considerable clinical and economic challenges [10]. Accurately determining the true prevalence of NAFLD is challenging due to inconsistent diagnostic criteria. This disease occurrence varies significantly across different geographical regions and ethnic groups, influenced by factors such as obesity rates, genetics, and socioeconomic status. For instance, Riazi et al. estimated that the overall prevalence of NAFLD among adults was 32% in 2016 or later, based on an analysis of data from 72 studies involving 1,030,160 individuals across 17 countries [11]. According to the United States Third National Health and Nutrition Examination Survey (NHANES III), the estimated prevalence of NAFLD as determined by ultrasonography is 34.0% in the United States [12]. In the Americas and South-East Asia, the prevalence of NAFLD surpasses 40% [13]. The pathogenesis of this condition can vary significantly among different populations based on their racial and ethnic backgrounds. Research indicates that race and ethnicity influence the development of NAFLD, in addition to other previously mentioned factors such as lifestyle choices, genetic predispositions, environmental influences, and socioeconomic status [14,15]. Nevertheless, there exists a notable gap in the literature regarding how these elements contribute to health disparities in NAFLD outcomes. Consequently, this review seeks to investigate emerging factors, including gut microbiota (GM) composition and epigenetic changes, and how they are related to racial and ethnic differences in this disease. By identifying distinct bacterial taxonomic profiles or epigenetic marks associated with phenotypic differences in NAFLD across various ethnic groups, healthcare professionals may have the opportunity to potentially reverse or slow disease progression. This could be achieved through modifications in diet or the use of pharmacological therapies that help restore balance, ultimately paving the way for the development of personalized and effective treatment approaches for NAFLD at both the individual and community levels.

## 2. Race/Ethnic Health Disparities in Susceptibility to NAFLD

Health disparities are defined as the avoidable differences in the burden of disease, injury, violence, or the ability to attain optimal health that are evident among socially disadvantaged populations [16]. These inequities manifest as differences in the rates of illnesses, health outcomes, or access to healthcare services among various groups. In the United States, health disparities constitute a significant public health concern, particularly affecting ethnic minority populations such as African Americans, American Indians and Alaska Natives, Asian Americans, and Hispanics. Several studies have revealed notable heterogeneities in the prevalence, incidence, and overall effects of NAFLD across diverse racial and ethnic groups [17,18,19]. Research on racial and ethnic disparities in NAFLD has predominantly concentrated on the Hispanic demographic in the United States. In this regard, Rich et al. performed a systematic review and meta-analysis encompassing 34 published studies up to 2016, which included a total of 368,569 participants. Their results indicated that the prevalence of NAFLD was markedly higher among the Hispanic population, at 22.9% (95% CI: 21.6–24.1%), in contrast to 13.0% (95% CI: 12.2–13.9%) in the Black population and 14.4% (95% CI: 14.0–14.8%) in the non-Hispanic White population [19]. This finding regarding Hispanic individuals aligns with earlier research that identified this ethnic group as having the highest prevalence of NAFLD, a condition frequently associated with elevated rates of obesity and diabetes within this population [20]. A research study by Huang et al., which analyzed data from the third NHANES, indicated that the prevalence of NAFLD was significantly elevated among the Hispanic population, reaching 37.0% [18]. In contrast, the prevalence among non-Hispanic Black individuals was lower, at 24.7%, while non-Hispanic White individuals showed a prevalence of 29.3% [18]. Other studies indicated that Mexican-origin adults are disproportionately impacted by NAFLD. Specifically, the prevalence of NAFLD among Mexican-origin adults stands at 42.8%, which is notably higher than the rates observed in non-Hispanic White adults at 30.6% and non-Hispanic Black adults at 21.4% [21]. The data indicate that the higher occurrence of NAFLD observed in Hispanic individuals, compared to those from other ethnic groups, distinctly underscores the presence of ethnic dissimilarities. Recently, Nguyen et al. published a study that reveals notable disparities in the outcomes of NAFLD among different racial and ethnic groups, based on longitudinal real-world data [22]. The results show that Black patients with NAFLD face the highest risk of both overall and non-liver-related mortality, followed by Hispanic patients, while Asian patients exhibit the lowest risk for all adverse outcomes when compared to White patients [22]. Furthermore, earlier research has shown that although the prevalence of NAFLD is lower among Black patients, they tend to experience more severe outcomes once the disease has established [23].

NAFLD is commonly found in Asian populations, particularly in countries like Japan, China, and India. In this context, the condition is associated with various factors, including urbanization, shifts in dietary habits, and increasing obesity rates. Additionally, these populations often exhibit insulin resistance and a greater proportion of visceral fat, which heightens the likelihood of developing NAFLD. In China, meta-analyses have indicated that the prevalence of NAFLD varies between 29.2% and 32.3% [17,24]. Moreover, NAFLD is diagnosed more often in males, with prevalence rates varying by region, ranging from 19.3% in Southwest China to 33.8% in the Northwest. A meta-analysis by Le et al. in 2019, which examined 182 studies involving a total of 2,385,999 individuals, estimated the prevalence of NAFLD in Asia to be 30.5% [25]. Furthermore, a recent meta-analysis by Riazi et al., encompassing 63 studies with 1,000,681 participants, found a prevalence of 31.6% for NAFLD in Asia [11]. This result is consistent with an earlier meta-analysis by Li et al., which reported a prevalence of 29.62% in the region [26]. A retrospective study in Japan (2014–2018) found that 25.8% of the general population had NAFLD, with men at 37.4% and women at 18.1% [27]. Nonetheless, to confirm the validity of these findings, future longitudinal and prospective studies are necessary, as retrospective cross-sectional studies do not establish causal relationships.

In Europe, various meta-analyses have evaluated the prevalence of NAFLD, estimating it to be between 23.7% [20] and 26.9% [28]. A more recent meta-analysis, which included 11 studies with a total of 15,062 participants, revealed that this prevalence has risen to 30.9% [29]. Additionally, another meta-analysis conducted by Riazi et al., which analyzed seven studies involving 14,111 individuals, reported a similar prevalence rate of 32.6% for NAFLD in Europe [11]. Among European countries, Turkey had the highest prevalence at 48.4%, followed by Italy at 38.2%. Germany, Portugal, and Spain reported prevalence rates between 25% and 27% [11]. Nonetheless, the prevalence rates of NAFLD across individual studies show considerable variation, ranging from 5% to 48%. This variability can be explained by factors such as the length of observation, geographical location, ethnic differences, study inclusion criteria, population characteristics, and the methods used to diagnose hepatic steatosis [30]. Overall, the results from these preliminary studies demonstrate that NAFLD impacts various racial and ethnic groups in distinct ways, highlighting the necessity for further research across different regions. Apart from the traditional contributors to ethnic inequities in NAFLD, research on GM composition and epigenetic mechanisms may also yield significant insights in this area.

## 3. The Potential Role of Gut Microbiota in Racial and Ethnic Disparities in NAFLD

The GM, which comprises bacteria, fungi, archaea, and viruses within the gastrointestinal tract, plays a vital role in human metabolism and the maintenance of the epithelial barrier’s integrity [31]. Alterations in the composition and diversity of GM can trigger the development and progression of several diseases by interfering with host metabolism and immune functions [32,33]. While gene–environment interactions have been a focal point in NAFLD research, recent studies indicate that dysbiosis, characterized by an imbalance in the microbiome, may play a significant role in metabolic disorders such as NAFLD. Additionally, the connection between NAFLD and changes in the gut microbiome composition, which notably affects metabolism, inflammation, and liver function, suggests that the microbiome could be a crucial element in the development of this condition [29,34]. Research has demonstrated that alterations in the composition and diversity of GM, especially an increase in proinflammatory bacteria, are correlated with the progression of NAFLD [35]. Specifically, individuals diagnosed with NAFLD frequently show higher concentrations of *Escherichia* and *Lactobacillus* in their stool, while the levels of *Coprococcus* and *Faecalibacterium* are generally lower [36]. A study by Rau et al. highlighted a notable reduction in *Alistipes onderdonkii* among NAFLD patients [37]. This observation is consistent with another investigation that reported a substantial decrease in *A. onderdonkii* in individuals with metabolic MetS, a condition closely related to NAFLD, when compared to a control group [38]. Additionally, an increased Bacteroidetes/Firmicutes (B/F) ratio is sometimes recognized as a dysbiotic marker associated with NAFLD [39]. Recent studies have yielded significant evidence indicating that variations in human microbiota are strongly associated with race and ethnicity, affecting disease patterns and health outcomes [40]. In the United States, research by Brooks et al. revealed that the occurrence of 12 distinct microbial genera and families showed consistent differences among African Americans, Asians/Pacific Islanders, Hispanics, and White people [41]. Furthermore, a study conducted in the Netherlands found that the differences in gut microbial compositions between African, Middle Eastern, or South Asian immigrants and Dutch individuals accounted for 5.7% of the observed variations among the 2084 participants [42]. In a cross-sectional analysis, Hullar and colleagues investigated the relationship between GM and the accumulation of liver fat among participants from diverse ethnic groups, including African American, Japanese American, White, Latino, and Native Hawaiian individuals, as part of the Multiethnic Cohort study [43]. The findings indicated that NAFLD was linked to alterations in bacterial composition and metabolic functions, suggesting that bacterial endotoxins may affect systemic inflammation related to liver fat in ways that vary by ethnicity. Furthermore, Setiawan and colleagues observed that NAFLD was the primary cause of chronic liver disease (CLD) across all five ethnic groups in the previous Multiethnic Cohort Study; however, the prevalence of NAFLD-related CLD varied among these populations [44]. A variety of factors have been recognized as impacting changes in the microbiome, including lifestyle choices, dietary habits, aging, coexisting health conditions, socioeconomic status, genetic predispositions, and the socioeconomic status of individuals. For instance, Amato et al. pointed out that specific variations in GM linked to structural inequalities are consistently correlated with social, economic, and structural stressors, which contribute to health disparities across various racial and ethnic groups [45].

The impact of diet on human health is extensively documented, especially regarding its influence on the composition of the gut microbiome [46]. In the previously mentioned Multiethnic Cohort, which encompasses five racial and ethnic groups [43,44], further analyses by Borrello K et al. revealed that diet quality had the most pronounced mediating effect on the ethnic differences in the prevalence of *Flavonifractor*, *Ruminiclostridium-5*, and *uncultured Ruminococcaceae* [47]. This indicates that the quality of diet and specific food choices may significantly contribute to the ethnic variations seen in gut microbiome composition and the related health disparities among different racial and ethnic groups. A study conducted by Zuo et al. investigated the human gut DNA virome in two different regions and among six ethnic groups in China, revealing notable differences in the virome among healthy individuals [48]. The researchers highlighted that several host factors significantly influence this variation, with geography, diet, medication, urbanization, and ethnicity being the most impactful. A separate study revealed that migrating from a non-Western nation to the United States leads to an immediate loss in the diversity and functionality of the gut microbiome, as US-associated strains and functions replace the native strains and functions [49]. These alterations were marked by reduced diversity, the elimination of native strains, a decrease in fiber degradation abilities, and a shift from *Prevotella* dominance to *Bacteroides* dominance [49]. Nevertheless, the authors propose that the alterations in the microbiome related to immigration are likely affected by various factors beyond diet and those linked to adapting to life in the U.S. To date, no specific gut microbiota “signature” has been reliably connected to any particular geographic area or ethnic group. These studies collectively indicate that the racial and ethnic differences in the prevalence of NAFLD are shaped by a complex interplay of various factors, including genetic predispositions, environmental conditions, and elements related to GM. The GM plays a crucial role in managing liver fat accumulation, inflammation, and metabolic functions. Alterations in gut microbiota composition across ethnic groups may help explain the differing susceptibility to NAFLD observed in certain populations. Consequently, additional research is necessary to explore the differences in the gut microbiome related to race and ethnicity, as these variations are likely influenced by a wide range of factors, including environmental and social conditions, cultural background, dietary habits, medication use, and access to food and healthcare, all of which play a role in health disparities.

Beyond research centered on NAFLD, diabetes, and obesity, emerging findings indicate that the GM is significantly linked to various other metabolic disorders, such as phenylketonuria (PKU), MetS, and atherosclerosis, with variations observed among different ethnic groups. For instance, the Uyghur population exhibits a higher prevalence of PKU compared to the Han population in China. In this context, Su et al. have shown a diminished presence of the *Bacteroides genus* in Uyghur patients with PKU, correlating with lower blood phenylalanine levels [50]. With regard to MetS, studies suggest that changes in the gut microbiome are linked to this condition, which presents unique compositions across various ethnicities [42,51,52]. Furthermore, the HELIUS (Healthy Life in an Urban Setting) study uncovered both ethnic-independent and ethnic-specific relationships between GM composition and MetS outcomes [53]. Further investigations within this multiethnic population have suggested that certain gut microbes may have a potential causal influence on cardiometabolic diseases, such as atherosclerosis, with effects that may differ across ethnicities [54]. Nonetheless, research on these metabolic disorders is still in its early stages and requires further investigation to improve understanding of the proposed connections between these disorders and GM across different ethnic groups. The studies mentioned above highlight the significant role of GM in metabolic diseases, which frequently exhibit shared pathological mechanisms, such as metabolic abnormalities, insulin resistance, inflammation, and oxidative stress. Research in this area can improve overall health outcomes for populations impacted by these conditions and help in identifying specific biomarkers that can lead to more accurate risk assessment and diagnosis within these groups. These biomarkers can also guide interventions that respect cultural dietary patterns and uncover how environmental and social stressors impact the gut microbiome in marginalized communities.

The GM is associated with a range of conditions beyond NAFLD, such as inflammatory bowel disease, cancer, respiratory illnesses, cardiovascular diseases, and even neurological disorders like depression and Alzheimer’s disease [55]. Given that the GM impacts various systems, including metabolic, immune, and neurological functions, a more in-depth investigation could uncover common biomarkers that signify disease risk or progression across different conditions. Ultimately, by understanding how gut microbial composition and metabolites influence health disparities among diverse ethnic groups, we can develop more targeted interventions that promote a healthier microbiome, thus enhancing overall health and mitigating the risk of both metabolic and non-metabolic diseases in these populations.

## 4. Epigenetic Determinants of Racial/Ethnic Disparity in NAFLD

Genetic, gender-related, and epigenetic factors may help explain part of the racial and ethnic disparities observed in NAFLD. Genetics may provide insights into certain racial and ethnic disparities. For example, variations in the patatin-like phospholipase domain-containing protein 3 (PNPLA3) gene have been found to be significantly linked to hepatic fat accumulation, with a higher prevalence observed among Hispanic individuals [56], particularly those of American descent [57]. This gene has also been strongly associated with alcoholic liver disease [58]. Subsequent research has indicated that the PNPLA3 rs738409 C > G single nucleotide polymorphism (SNP) is more frequently found in Hispanic populations, contributing to elevated rates of NAFLD [59,60]. Gender also may interact with racial and ethnic factors to contribute to the epidemiology and clinical progression of NAFLD. In systematic reviews and meta-analyses comprising 54 studies and 62,239 participants, consisting of 35,119 women and 27,120 men, Balakrishnan et al. identified significant gender disparities in NAFLD prevalence, indicating that women had a 19% lower risk of NAFLD than men in the general population [61]. Additionally, research has demonstrated that racial and ethnic variations in NAFLD prevalence are affected by gender, with Mexican American men at a heightened risk for developing the most severe forms of the disease [62]. The National Health and Nutrition Examination Survey (NHANES) conducted in 2017–2018 revealed that NAFLD prevalence increases with age, being more significant in men than in women, and is particularly elevated among Hispanic individuals compared to non-Hispanic White people [63]. However, once diagnosed with NAFLD, women are equally likely to develop nonalcoholic steatohepatitis (NASH) and face a higher risk of advanced fibrosis [61]. Epigenetic mechanisms, which mediate gene–environment interactions, are likely to be crucial in influencing these disparities. The subsequent discussion will explore epigenetic alterations’ impact on the risk and progression of NAFLD across various populations.

Epigenetics involves a variety of modifications to the genome and its associated proteins, which primarily include DNA methylation, alterations to histone tails, nucleosomal remodeling, and changes in transcription and translation mediated by non-coding RNAs (ncRNAs). These modifications can affect gene expression patterns without changing the underlying DNA sequence. Additionally, epigenetic modifications play a crucial role in numerous biological processes and significantly influence gene expression and function related to metabolic diseases, making them potential biomarkers for the early detection of such conditions. It is recognized that epigenetic mechanisms can be modified in response to various factors, including genetic predispositions, environmental influences, lifestyle choices, socioeconomic status, and diet [64]. Each of these factors has been associated with metabolic diseases like NAFLD, revealing notable disparities among different racial and ethnic groups.

Among the various epigenetic mechanisms, DNA methylation is particularly notable for being one of the most extensively studied and stable processes. Early investigations indicated significant differences in DNA methylation levels at specific CpG dinucleotides between African American and Caucasian infants [65]. Subsequent research has indicated that DNA methylation patterns vary according to race and/or ethnicity [66]. More recently, considerable differences in DNA methylation across the genome have been observed when comparing South Asians to Europeans [67]. The interplay between DNA methylation levels and factors such as genetic and environmental influences can affect health outcomes. For instance, research has revealed a notable interaction effect concerning global DNA methylation levels, race/ethnicity, socioeconomic status, and the severity of asthma [68]. Emerging evidence in cancer research indicates that there are racially specific patterns of DNA methylation present in normal tissues, along with changes in methylation that occur during oncogenesis. Notable differences in the prevalence of DNA methylation have been observed in prostate tissue samples from African American individuals compared to those from Caucasian individuals across various genes, suggesting that these differences may contribute to the disparities associated with prostate cancer [69]. With respect to NAFLD, however, there is a significant lack of data in populations that are racially, ethnically, and socioeconomically diverse and how they affect health disparities. The following paragraph will examine the existing knowledge on the epigenetic factors that could play a role in the racial and ethnic disparities observed in NAFLD.

Experimental research indicates that changes in DNA methylation patterns play a significant role in the development of NAFLD [70,71,72]. To identify the DNA methylation profile in peripheral blood associated with liver fat, Ma et al. conducted epigenome-wide association studies (EWASs) with a sample of 3400 individuals of European descent, 401 of Hispanic descent, and 724 of African descent from four population-based cohorts [73]. The results of this study revealed a strong correlation between the DNA methylation profile from peripheral blood and the accumulation of hepatic fat, indicating a possible causal relationship of this epigenetic mechanism in NAFLD. Furthermore, this research identified 22 CpG sites associated with hepatic fat, especially among individuals of European descent. Notably, the hypomethylation of a specific CpG site within the long intergenic nonprotein coding RNA 649 (LINC00649) gene was significantly associated with NAFLD and an increased risk of developing new-onset T2DM. These findings indicate that racial differences may play a role in the regulation of DNA methylation during the advancement of liver diseases [73]. Additionally, alterations in DNA methylation associated with fatty liver and metabolic disorders have been detected in a varied group of pre-adolescent children. In a comprehensive epigenome-wide association study focused on liver fat accumulation and liver damage within this demographic, Moylan et al. identified two particularly significant differentially methylated regions: one positioned downstream of the ZNRD1 (Zinc ribbon domain-containing 1) gene, also known as RNA polymerase I subunit N, and the other located in the promoter region of the GLTPD2 gene, which encodes the glycolipid transfer protein domain containing 2 [74]. The metabolic hallmark of obesity is NAFLD, and obesity is known to be a major risk factor for this condition. In this context, a recent investigation highlighted the racial differences in the methylation patterns of the nuclear respiratory factor 1 (NRF1), fat mass and obesity-associated (FTO), and leptin receptor (LEPR) genes among children with obesity [75]. Furthermore, a cross-sectional study conducted by Song et al. revealed notable differences in genome-wide differential methylation patterns between Japanese American and European American women aged 60 to 65. These differences were particularly pronounced in genes potentially linked to liver function and diseases, which are frequently associated with lifestyle modifications and socioeconomic disparities [76]. Epigenetic mechanisms have been identified as a significant contributor to the development of MASLD. An increasing amount of research suggests that maternal conditions such as pre-pregnancy obesity, diabetes, weight gain during pregnancy, and gestational diabetes, are associated with heightened fatty acid levels in the fetus, which may predispose children to MASLD [77]. Furthermore, the prevalence of gestational diabetes is reported to be two to three times higher among Hispanic populations compared to the general population, which may lead to a higher occurrence of MASLD in these communities [78].

Histone post-translational modifications are another important epigenetic regulation mechanism that has attracted attention due to their association with diseases and their potential as therapeutic targets. Sirtuin 1 (SIRT1) is an NAD(+)-dependent histone deacetylase that functions as a post-translational regulator for the deacetylation of acetyllysine. SIRT1 deacetylates histones and non-histones to regulate various physiological functions [79]. This multi-functional protein exerts a predominant effect on regulating hepatic lipid metabolism and NAFLD development by deacetylating its targets. In NAFLD patients, hepatic SIRT1 mRNA levels are downregulated and negatively associated with hepatic steatosis and fibrosis [80,81]. Other studies have shown that SIRT1 has a significant impact on liver lipid metabolism, oxidative stress, and inflammation by means of epigenetic modifications that help mitigate the progression of fatty liver disease [82]. Notably, hepatic SIRT1 is activated during caloric restriction and intermittent fasting, which enhances the NAD+/NADH ratio. The activation of SIRT1 offers protective effects against hepatosteatosis, glucose intolerance, and fibrosis induced by a high-fat diet [83]. In addition, SIRT1 contributes positively to the regulation of hepatic lipid metabolism, the management of oxidative stress in the liver, and the prevention of liver inflammation by deacetylating various transcriptional regulators, thereby mitigating the progression of fatty liver diseases [82]. Several investigations have indicated a link between the dysregulation of SIRT1 and the advancement of NAFLD via mechanisms involving DNA methylation and histone modifications [79,84]. Others have reported that interactions between SIRT1 and p53 may influence the regulation of adipocytokines and immunometabolism, which are significant factors in NAFLD, obesity, and neurodegeneration [84]. In an elderly population of Han Chinese, research demonstrated that the relationship between SIRT1 genetic variation and T2DM is modulated by dietary habits [85]. Furthermore, studies have shown that Asians exhibit a higher incidence of hepatic steatosis and insulin resistance compared to Caucasians, suggesting a potential increased risk for NAFLD within this ethnic group [86,87].

While these studies have identified notable epigenetic differences among various populations that could explain the disparities in adiposity, disease progression, severity, and outcomes associated with NAFLD, investigating the interaction between the GM and epigenetics could provide valuable insights into NAFLD disparities across diverse racial and ethnic groups. Therefore, it is essential to conduct further studies that consider the interplay between epigenetics and GM and their interaction with host physiology.

**Figure 1:**
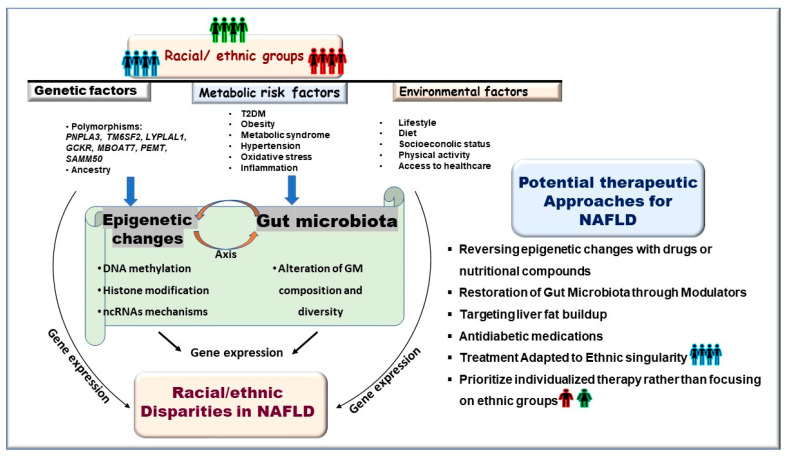
Schematic illustration of potential contributors to racial and ethnic disparities in NAFLD. This includes the effects of numerous elements such as genetic variations, environmental factors, nutrient signals, metabolic risk factors, lifestyle decisions, and socioeconomic status on the gut microbiota–epigenetic axis. The subsequent changes in the composition and diversity of the gut microbiome, along with alterations in the genetic landscape, significantly affect gene expression, potentially leading to racial and ethnic inequalities in NAFLD.

Both epigenetics and GM may act as mediators between environmental exposures and health outcomes, as depicted in Figure 1. They are influenced by numerous factors, including dietary habits, genetic predispositions, lifestyle choices, and socioeconomic conditions. These factors can lead to changes in GM composition and function, which may significantly affect the pathogenesis of NAFLD through epigenetic pathways. Conversely, epigenetic mechanisms can also mediate the relationship between GM and the host. The emerging bidirectional axis between the epigenome and microbiome provides significant insights into enhancing our understanding of host–microbe interactions and their coevolution [88,89]. Notably, the interplay between epigenome and microbiome is linked to the pathophysiology of NAFLD, indicating potential new therapeutic strategies [90]. From these observations, it can be inferred that the epigenome–microbiome axis may impact the burden of NAFLD and contribute to racial and ethnic disparities. Thus, a thorough understanding of genetic and non-genetic exposures, along with bidirectional gut microbiota–epigenetic interactions, is crucial for guiding treatment decisions related to NAFLD.

## 5. Therapy for NAFLD: Exploiting the Reversibility of Gut Microbiota Composition and Epigenetic Changes for Potential Treatment

### 5.1. Restoring Dysbiosis in Gut Microbiota Alleviates NAFLD

There is substantial evidence establishing a relationship between the gut, its microbiota, and NAFLD, referred to as the gut–liver axis [39]. Recent progress in microbiome research is providing new avenues to target the gut microbiome and their metabolites as novel treatments for metabolic disorders. Among the various strategies to combat gut dysbiosis, prebiotics, probiotics, fecal microbiota transplantation (FMT), dietary changes, and antibiotics stand out as the most promising. These methods have shown potential in restoring the microbial balance in the gut and may even reverse certain features of NAFLD [32,91,92].

Recent research underscores the significance of probiotics and prebiotics in promoting gastrointestinal health. The focus of these studies has largely been on different species of *Lactobacillus*, *Bifidobacterium*, and *Streptococcus* as probiotic agents, as well as *fructooligosaccharides* serving as a source of prebiotics [93]. Probiotics play a vital role in preserving or reestablishing the equilibrium of gut microbiota, a key element in mitigating NAFLD and promoting the overall well-being of the individual [94]. Research indicates that both *Lactobacillus* and *Akkermansia* may effectively diminish liver injury, rectify metabolic disorders in the host, and counteract the disruptions in gut microbiota caused by a high-fat diet (HFD) [95,96]. The administration of the probiotic *Lacticaseibacillus rhamnosus GG* (LGG) has been found to reverse decreased FGF21 expression, enhance adipose tissue production of adiponectin (ADPN), and diminish hepatic fat accumulation and inflammation in murine models [97]. Supplementation with probiotics and prebiotics has shown potential in lowering the liver enzymes alanine aminotransferase (ALT) and aspartate aminotransferase (AST) in patients with liver damage [98,99]. These interventions aim to modulate the gut–liver axis and restore disrupted gut microbiota, which is crucial in the treatment of NAFLD [100]. A review of significant randomized clinical trials involving probiotics and prebiotics in patients with hepatic steatosis has been conducted recently [101]. Besides a reduction in endogenous toxins and microbial translocation, improvements in NAFLD after treatment with probiotics and prebiotics were demonstrated to be associated with weight loss [102,103]. Certain probiotic strains have shown potential in reducing body weight and improving insulin sensitivity in obese patients, enhancing glycemic control in diabetic patients, and providing therapeutic benefits for those with NAFLD [104,105]. These results collectively imply that probiotics and prebiotics have potential for enhancing gut health and reducing the risk of NAFLD.

The gut microbiota composition can also be modulated by fecal microbiota transplantation (FMT) [106], a procedure that involves the transfer of fecal material from a healthy donor to a patient with a disease, aiming to restore the diversity and composition of the microbiota [107]. FMT has demonstrated significant clinical effectiveness in treating Clostridium difficile infections (CDIs) and shows potential in addressing other conditions associated with changes in gut microbiota, such as inflammatory bowel disease, obesity, MetS, and functional gastrointestinal disorders [108,109]. It has been established that dysbiosis within the intestinal microbiota may contribute to metabolic disorders, including obesity, diabetes, and fatty liver disease [110]. Currently, only small studies and Phase I trials of FMT are available. However, in addition to its application in CDIs, several encouraging randomized controlled trials have assessed gut microbiota therapy for NAFLD, evaluating the effectiveness and feasibility of such treatments in this context [111,112,113,114]. FMT, potentially delivered through oral capsules, could become a useful therapeutic strategy for the management of metabolic diseases [115]. While FMT is proven to be highly effective for Clostridium difficile infections, its application in other diseases remains under investigation and requires optimization of FMT regimen for each disease, establishing efficacy and safety with larger clinical trials. From the perspective of drug development in racial and ethnic populations using the approach of GM, a study emphasized the possible therapeutic benefits of gut microbiota in MetS [116]. It was found that FMT from lean donors to obese Dutch males with MetS resulted in a temporary enhancement of insulin sensitivity after six weeks, in contrast to those who received their own fecal microbiota. These findings underscore the potential of gut microbiota modulation in addressing key metabolic disturbances. Given that 90% of individuals with NAFLD have at least one risk factor of MetS, and 33% exhibit all the syndrome’s features, such interventions could hold particular promise for this high-risk population.

Considering the connection between diet and the composition of GM, researchers have investigated dietary interventions as a means to improve gut dysbiosis, which may hold potential for treating NAFLD. Several studies have highlighted the effectiveness of various dietary approaches, such as the Mediterranean diet, ketogenic diet, intermittent fasting, and high-fiber diets, in enhancing gut microbial composition and reducing the pathogenesis and progression of MASLD [117]. Adhering to a Mediterranean diet (Med-diet) is associated with a healthier cardiometabolic profile. In this respect, a randomized pilot trial conducted in Puerto Rico by Mattei et al. aimed to assess the efficacy of a culturally tailored Mediterranean-like diet on cardiometabolic risk factors [118]. The study found that the intervention was culturally appropriate, acceptable, accessible, and feasible for adults in Puerto Rico (clinicaltrials.gov registration #NCT03975556). The preliminary results suggested that a culturally appropriate Med-diet could improve cardiometabolic health and diet quality, supporting its wider implementation in ethnic and multiethnic clinical and population-wide disease-prevention programs. The above-mentioned population-based cohort studies, investigating racial and ethnic disparities in the pathogenesis of NAFLD, certainly hold promise as potential therapeutic avenues, and may serve to determine which ethnic groups with NAFLD would benefit from specific interventional therapies. Microbiota-targeted therapies offer a promising avenue for treating NAFLD, with potential benefits for Hispanic, African American, and Asian populations. However, further research is needed to understand ethnic-specific responses to these therapies and to ensure equitable representation in clinical trials. Continued efforts to include diverse populations in research will be crucial for developing effective, personalized treatments for NAFLD.

### 5.2. Reversibility of Epigenetic Mechanisms

The regulation of gene expression through epigenetic modifications has emerged as a fascinating area of research, offering new possibilities for the treatment of NAFLD. A variety of epigenetic enzymes play a crucial role in maintaining epigenetic balance by either adding (writers) or removing (erasers) epigenetic marks. DNA methylation, recognized as the first epigenetic mechanism, is facilitated by DNA methyltransferases (DNMTs). Furthermore, histone acetylation and deacetylation are carried out by histone acetyltransferases (HATs) and histone deacetylases (HDACs), respectively, while histone methylation is conducted by histone methyltransferases (HMTs). When these enzymes are dysregulated, it leads to abnormal epigenetic modifications. Importantly, these changes are dynamic and reversible, as lifestyle and environmental influences can alter epigenetic patterns throughout a person’s life, allowing for potential adjustments. As a result, modifications in genes and proteins related to epigenetics may offer promising therapeutic strategies in clinical settings [119,120]. Recent progress in epigenetics has led to the development of numerous therapies aimed at specific epigenetic mechanisms for a range of diseases, especially cancer [121,122]. At present, the U.S. FDA has not approved any epigenetic drugs for the treatment of nonalcoholic fatty liver disease (NAFLD). Nevertheless, many research efforts are currently investigating potential epigenetic therapeutic options for this condition and its related complications.

#### 5.2.1. Potential Epigenetic Drugs in Clinical Trials for NAFLD

Research is currently underway to evaluate certain experimental drugs that may effectively target specific epigenetic alterations, including histone deacetylase inhibitors (HDACis) and DNA methyltransferase inhibitors (DNMTis), which could impact the development of NAFLD. Some studies have indicated that histone acetylation might serve as a viable target for NAFLD treatment. Tannic acid (TA), identified as a histone acetyltransferase inhibitor (HATi), has demonstrated the ability to prevent NAFLD by inhibiting lipid accumulation in vivo and reducing the mRNA expression of genes linked to lipogenesis [123]. On a mechanistic level, TA has been shown to disrupt the binding of p300 to the sterol regulatory elements (SREs) in the promoters of the FASN and ATP-citrate lyase (ACLY) genes, leading to a decrease in the acetylation of H3K9 and H3K36 [123]. Furthermore, the beneficial impacts of polyphenols on NAFLD have been explored in numerous clinical studies. For instance, curcumin, a polyphenolic compound derived from turmeric, which is recognized in traditional Chinese medicine [124], has demonstrated a reduction in total triglycerides and waist circumference in individuals with NAFLD, as highlighted in a recent systematic review and meta-analysis [125]. A short-term daily regimen of curcumin over eight weeks also resulted in a decrease in liver fat and serum levels of ALT and AST among NAFLD patients [126]. Specifically, a daily dosage of 70 mg of curcumin led to a 78.9% reduction in liver fat content for those with NAFLD, compared to a mere 27.5% improvement in the placebo group. Curcumin is noted for its various pharmacological effects, including the inhibition of p300-specific histone acetyltransferase (HAT) activity [127] (Table 1).

Clinical studies have demonstrated that curcumin offers significant therapeutic benefits for conditions such as T2DM, obesity, and NAFLD. A noteworthy clinical trial (NCT02908152, available at https://clinicaltrials.gov/ (accessed on 7 March 2025)) is currently in Phases 2/3 and has shown that a daily dosage of 1.5 g of curcumin in patients with NAFLD led to a decrease in hepatic fibrosis and steatosis, along with a reduction in inflammation in these patients [128]. Additionally, another clinical trial (NCT03864783) is underway (refer to Table 1), which aims to explore the impact of curcumin on liver fat content in obese individuals suffering from NAFLD (https://clinicaltrials.gov/search?intr=NCT03864783, accessed on 25 March 2025).

Similarly, resveratrol’s ability to decrease liver fat accumulation caused by diet through enhanced fatty acid oxidation and reduced lipogenesis suggests its potential as a therapeutic agent for preventing NAFLD [129]. Research has demonstrated that resveratrol activates SIRT1, an enzyme recognized for its positive impact on hepatic lipid metabolism [82]. In this context, a Phase 2/3 clinical trial [ClinicalTrials.gov NCT02030977] involving 50 NAFLD patients found that administering either a 500 mg resveratrol capsule or a placebo for 12 weeks led to improvements in inflammatory biomarkers among those with NAFLD [130]. Conversely, a separate placebo-controlled clinical trial (NCT01446276, ClinicalTrials.gov) that evaluated higher doses of resveratrol found no significant impact on insulin-mediated VLDL-TG secretion, oxidation, or clearance rates in obese men suffering from NAFLD [131]. Nevertheless, the current body of evidence remains inadequate for establishing the effectiveness of resveratrol in treating NAFLD, as highlighted by various inconsistencies in the existing scientific literature [132]. These discrepancies may arise from several factors, including variations in the study population, methodological flaws in the clinical trial designs, limited sample sizes, or the specific dosages and administration methods employed.

Vupanorsen (AKCEA-ANGPTL3-L Rx) is an N-acetyl galactosamine-conjugated antisense oligonucleotide designed to target the liver and specifically inhibit the synthesis of angiopoietin-like 3 (ANGPTL3) protein. It is currently undergoing a Phase 2 clinical trial (NCT03371355). The findings from this study indicate that a total monthly dose of 40–80 mg administered subcutaneously to patients with elevated triglycerides, T2DM, and NAFLD resulted in dose-dependent decreases in ANGPTL3, TG, ApoC-III, VLDL, non-HDL cholesterol, and total cholesterol, without any observed reductions in liver fat or HbA1c levels. The treatment demonstrated a favorable safety and tolerability profile [133]. Furthermore, incorporating insights from ethnic studies into the design of NAFLD treatments is essential for creating therapies that are effective for a wide range of populations.

#### 5.2.2. Potential Epigenetic Targets for the Treatment of NAFLD

Epigenetic enzymes are increasingly being recognized in the biological context of NAFLD. For instance, the histone methyltransferase Suv39h2 has been identified as a contributor to the pathogenesis of NASH by enhancing the inflammatory response in hepatocytes and macrophages. The absence of Suv39h2 in knockout mice has been shown to protect these animals from developing NASH, indicating that Suv39h2 may serve as a promising target for the creation of new therapeutic strategies [134]. Considering the essential function of epigenetic enzymes in the regulation of inflammation linked to NASH progression, epigenetic inhibitors could hold significant promise for the exploration and development of innovative treatments for this condition. Decitabine, known scientifically as 5-aza-2′-deoxycytidine, is recognized as the most potent DNMT inhibitor. In relation to NAFLD, both 5-aza-2′-deoxycytidine and curcumin have demonstrated the ability to reverse DNA methylation of peroxisome proliferator-activated receptor-α (PPAR-α), a factor implicated in the development of NAFLD [135] (refer to Table 2). Additionally, HDACi may represent promising therapeutic options for metabolic disorders, although they have not yet been approved by the U.S. FDA.

Givinostat functions as an inhibitor of lysine deacetylase and has demonstrated efficacy in improving NASH by reducing the levels of inflammatory cytokines such as IL-6, IL-1β, and TNF-α [136] (Table 2). Additionally, this compound has been recognized for its significant role in altering chromatin structure and counteracting pathological processes associated with Duchenne muscular dystrophy [137]. SIRT1, an NAD+-dependent deacetylase, is linked to a variety of diseases, including cancer, hypertension, and diabetes. Increasing evidence indicates that SIRT1 plays a role in the progression of NAFLD. In patients with NAFLD, hepatic SIRT1 mRNA levels are found to be downregulated and inversely correlated with hepatic steatosis and fibrosis [80,81]. The suppression of SIRT1 expression or activity accelerates the advancement of NAFLD [138]. Therefore, SIRT1 presents a potential therapeutic target for NAFLD. Additionally, SIRT1 is involved in mediating the effects of exenatide in reducing hepatic steatosis, indicating that the glucagon-like peptide-1 (GLP-1) receptor agonist may be a promising treatment option for NAFLD [139]. A recent investigation revealed that resveratrol inhibits insulin resistance and hepatic steatosis induced by HDF by downregulating the activation of SIRT1 mediated by miR-34a [140]. Quercetin, the most prevalent flavonoid in our diet, has been shown in previous research to offer potential therapeutic effects against NASH caused by HFD in gerbils [141]. When used in combination with metformin, quercetin was found to diminish hepatosteatosis by promoting autophagy via the cCAMP/AMPK/SIRT1 signaling pathway [142]. The histone demethylase KDM1A has been identified as a contributor to hepatic steatosis and inflammation by enhancing chromatin accessibility in NAFLD [143]. These results indicate that the KDM1A enzyme could serve as a potential therapeutic target in this area. Based on these preliminary studies, all of these compounds, summarized in Table 2, show promising potential as epigenetic targets for the treatment of NAFLD.

**Table 2:** Potential epigenetic-targeted drugs offering promising prospects for the treatment of NAFLD.

Drug	Class	Mechanism of Action	Ref.
5-Aza-CdR + Curcumin	DNMTi	Reverse PPAR-α DNA methylation involved in NAFLD	[135]
Givinostat	HDACi	Potential drug for treatment of NASH and liver fibrosis	[136]
GLP-1RAs	SIRT activator	Sirt activator ameliorates hepatic steatosis and may serve as a potential drug for NAFLD	[139]
Resveratol	SIRT1 activator	Prevents HFD-induced insulin resistance and hepatic steatosis by suppressing miR-34a-induced activation of SIRT1	[140]
Quercetin	SIRT1 activator	Significant therapeutic benefits for the prevention of NASH	[141]
Metformin+ Quercetin	SIRT1 activator	Alleviate hepatic steatosis by activating autophagy through the cAMP/AMPK/SIRT1 pathway	[142]
Target	KDM1A	Histone demethylase KDM1A promotes hepatic steatosis and inflammation by increasing chromatin accessibility in NAFLD	[143]

Abbreviations: AMPK, AMP-activated protein kinase; ASO: oligonucleotide antisense; cAMP, cyclic adenosine monophosphate; HATi; histone acetyltransferase inhibitor; ID: identification; KDM1A, lysine-specific histone demethylase 1; miR-34a, microRNA 34a; NAFLD: nonalcoholic fatty liver disease; Sirtuin 1; SIRT1-activating compound; STAC, sirtuin-activating compounds; T2DM: type 2 diabetes mellitus.

#### 5.2.3. Development of Epigenetic Biomarkers for NAFLD

Epigenetic biomarkers play a vital role in the early identification and prognosis of metabolic diseases. The influence of gene expression modulation through changes in DNA methylation within promoter regions is well established, particularly regarding disorders related to NAFLD. Notably, the methylation status of the mitochondrially encoded NADH dehydrogenase 6 (MT-ND6) has been closely associated with the severity of NAFLD, as highlighted by Pirola et al. [144]. Additionally, Johnson et al. [145] discovered seven CpG sites, including cg09822959 and cg19686543, that correlate with liver fibrosis and may represent valuable biomarkers for this condition. Furthermore, circulating miR-135a-3p present in serum extracellular vesicles, along with circulating miR-33a, is gaining recognition as potential biomarker for NAFLD [146,147]. A number of other specific microRNAs, including miR-21, miR-29, miR-33, miR-34, miR-122, miR-146, and miR-223, have been associated with the development and progression of NAFLD, indicating their possible roles as biomarkers or therapeutic targets, as discussed in a recent review by Mahdizadeh et al. [148]. Circular RNAs (circRNAs) represent a category of non-coding RNAs distinguished by their covalently closed single-stranded loop formations, lacking both a 5′ cap and a 3′ poly (A) tail [149]. These circRNAs show considerable promise as potent biomarkers for NAFLD. An increasing amount of research suggests that a particular set of relatively abundant circRNAs is essential in the cellular processes that contribute to the onset and advancement of NAFLD. For instance, the levels of cytoplasm-localized circRNAs, such as circRNA_0046367, circRNA_0046366, hsa_circ_0048179, circScd1, hsa_circRNA_021412, and circRNA_0001805, were significantly diminished in both cellular and animal models of NAFLD, as well as in individuals diagnosed with the condition [150]. Restoring these circRNAs has been demonstrated to reduce oxidative stress, hepatic steatosis, mitochondrial dysfunction, and the overall severity of the disease [151,152]. Furthermore, the overexpression of circRNA_0001805 through a nanodrug delivery system markedly decreased lipid accumulation and inflammation in NAFLD by targeting miR-106a-5p/miR-320a [153]. However, there is a lack of studies investigating the relationship between epigenetic mechanisms and the racial and ethnic disparities observed in NAFLD. While some research has addressed ethnic variations in DNA methylation, there has been little focus on histone modifications or the regulatory roles of non-coding RNAs on gene expression that could influence NAFLD disparities. Consequently, it is crucial for the research community to direct its efforts toward this area, as such investigations could significantly improve drug development for NAFLD.

The previously mentioned general therapeutic strategies, along with population-based cohort studies that explore the potential of epigenetic drugs in the development of NAFLD, certainly present promising therapeutic options. These approaches may help identify which ethnic groups suffering from NAFLD could benefit from specific interventional therapies. Mechanistically, the emerging treatments may contribute to reducing disparities by focusing on mechanisms that are particularly relevant to certain ethnic groups. For instance, in addition to molecular genetic profiling, epigenetic markers such as DNA methylation are affected by diet, socioeconomic status, stress, and environmental exposures, all of which differ across racial and ethnic groups.

## 6. Perspectives on Future Research and Conclusions

In recent decades, there has been considerable advancement in the examination of ethnic disparities concerning susceptibility to NAFLD and its related complications. This advancement is partly due to comprehensive cohort studies, such as the Healthy Life in an Urban Setting (HELIUS) study in Europe and the patient cohorts from the National Health and Nutrition Examination Survey (NHANES) in the United States [154,155,156]. These studies have provided valuable scientific insights into how ethnicity influences the primary factors contributing to the global burden of NAFLD. Nevertheless, despite these developments, the incorporation of race and ethnicity into clinical decision-making processes remains largely unachieved, primarily due to a deficiency of relevant studies and scientific evidence that would support this approach for the following reasons: (i) The complex and varied nature of NAFLD presents considerable difficulties in identifying the factors that contribute to the increased vulnerability of certain ethnic groups to the disease and its related complications. Moreover, the interaction of various elements such as genetic factors, epigenetic influences, environmental circumstances, lifestyle choices, healthcare accessibility, and cultural aspects adds to the challenge of understanding how these factors result in disparities in NAFLD. (ii) Research conducted in diverse, frequently limited populations can result in differing rates of prevalence, risk factors, and disease outcomes, as these studies may not adequately consider the multitude of factors that lead to disparities. Additionally, the designs and methodologies used may fail to effectively represent the complexities of these interactions, complicating data interpretation. Consequently, there is an urgent requirement for comprehensive, long-term cohort studies that monitor ethnic populations over time. These research efforts would offer essential insights into the ethnic differences in the onset, progression, and outcomes of the disease. (iii) Insufficient data and human clinical trials impede our comprehension of the pathogenesis of NAFLD and the associated racial and ethnic disparities, thereby hindering the advancement of effective treatments tailored for various populations. Advancing fair healthcare and improving access and delivery for all racial and ethnic communities is crucial for attaining health equity and enhancing outcomes for marginalized populations. Furthermore, it is crucial to establish educational programs and initiatives aimed at fostering behavioral changes in ‘at-risk’ groups, with an emphasis on identified modifiable factors. (iv) The epigenetic basis for racial and ethnic disparities in NAFLD is gaining increasing recognition. The early identification of epigenetic alterations could facilitate the development of an epigenetic signature tailored to specific ethnic groups. When integrated with other biomarkers, this signature has the potential to enhance the management of NAFLD and its associated complications across various populations. However, the practical application of this concept may be challenging, as environmental factors consistently affect the epigenome of both entire ethnic groups and individual members within those groups. Consequently, targeting the human epigenome or using epigenetic profiling as a tool for reducing ethnic health disparities would require more fine-tuned approaches to capture the diversity within ethnic populations, their individual characteristics, lifestyles, and the various environments they experience. (v) Additionally, the gut microbiota is increasingly acknowledged as a possible factor contributing to the ethnic and racial differences seen in NAFLD. While treatment options such as probiotics, prebiotics, and FMT demonstrate promise in restoring a healthy microbiome, their effectiveness is still uncertain. The majority of research has been conducted using animal models, highlighting the need for well-structured randomized clinical trials in humans before these treatments can be recommended. Recent studies indicate that fluctuations and instability in the gut microbiome may persist for many years in individuals with NAFLD, potentially even arising before the onset of NAFLD and T2DM [157]. This underlying instability could influence the results of short-term studies that investigate the impact of diet on gut microbiota, particularly among diverse ethnic populations. (vi) The link between specific changes in microbial communities and the development of NAFLD is not well understood. Most current studies are cross-sectional, making it difficult to determine whether changes in the microbiota are a cause or a result of NAFLD. To better understand this relationship, longitudinal cohort studies are necessary to track individuals over time and investigate how fluctuations in the microbiota are associated with the onset or progression of NAFLD.

To sum up, although there is currently no singular treatment aimed specifically at addressing racial and ethnic disparities in NAFLD, innovative approaches that integrate GM, genetic/epigenetic knowledge, personalized medicine, metabolic therapies, and community health initiatives show promise. Future research should extend beyond merely identifying these disparities to focus on discovering unique biological markers or specific characteristics that may have significant clinical relevance. In this context, the identification of genetic and epigenetic biomarkers, along with specific signatures of gut microbiota, could be crucial for more precise prediction and treatment of NAFLD. Additionally, mechanistic studies are essential to reveal the underlying causes of ethnic differences in NAFLD risk. These approaches may yield important insights into the elements contributing to the high rates of NAFLD and its associated complications, enabling earlier diagnosis and assessment of disease progression in minority ethnic populations, ultimately aiding in the prevention or mitigation of health disparities.

## Figures and Tables

**Table 1:** Epigenetic-based drugs for NAFLD treatment registered on ClinicalTrials.gov (accessed on 26 March 2025).

Clinical Trial ID/Phase	Class	Compound	Clinical Trial.gov/Primary Outcome
**NCT02908152**	HATi	Curcumin	https://clinicaltrials.gov/study/NCT02908152 (accessed on 28 April 2025)
Phase 2/3			The effect of curcumin supplement on metabolic factors and hepatic fibrosis in nonalcoholic fatty liver patients. Curcumin may reverse PPARα methylation and suppress FOXO1 acetylation.
**NCT03864783**	HATi	Curcumin	https://clinicaltrials.gov/search?intr=NCT03864783 (accessed on 28 April 2025)
Not applicable			The effect of curcumin on liver fat content in obese subjects with NAFLD. Curcumin reduces TG in NAFLD, inhibits NAFLD progression.
**NCT04315350**	HATi	Curcumin	https://clinicaltrials.gov/study/NCT04315350 (accessed on 28 April 2025)
Not applicable			The effect of curcumin on the development of prednisolone-induced hepatic insulin resistance.
NAFLD/Insulin resistance			
**NCT01446276**	STAC	Resveratrol	https://www.clinicaltrials.gov/search?intr=NCT01446276 (accessed on 28 April 2025)
Not applicable			Long-term investigation of resveratrol on fat metabolism in obese men with NAFLD.
**NCT02030977**	STAC	Resveratrol	https://www.clinicaltrials.gov/search?intr=NCT02030977 (accessed on 28 April 2025)
Phase 2/3			The effects of resveratrol supplement on lipid profile, liver enzymes, inflammatory factors, and hepatic fibrosis in patients with nonalcoholic steatohepatitis.
**NCT05419765**	-	-	https://clinicaltrials.gov/search?intr=NCT05419765 (accessed on 28 April 2025)
			Nonalcoholic fatty liver disease: crosstalk between genetic predisposition and epigenetic lysosomal acid lipase activity reduction in blood, plasma, and platelets.
**NCT02148471**	-	-	https://www.clinicaltrials.gov/search?intr=%20NCT02148471 (accessed on 28 April 2025)
			Fatty acids, genes, and microbiota in fatty liver. Study of gene expression and regulation by miRNA. Role of microbiota composition in NAFLD.
**NCT03371355**	ASO	-	https://www.clinicaltrials.gov/search?intr=NCT03371355 (accessed on 28 April 2025)
Phase 2			Study of ISIS 703,802 in participants with hypertriglyceridemia, T2DM, and nonalcoholic fatty liver disease.
**NCT03915002**	-	-	https://clinicaltrials.gov/search?intr=NCT03915002 Observational (accessed on 28 April 2025)
			Integrated approaches for identifying molecular targets in liver disease.

Abbreviations: ASO: oligonucleotide antisense; FOXO1, Forkhead Box O1; ID: identification; HATi; histone acetyltransferase inhibitor; NAFLD: nonalcoholic fatty liver disease; PPAR-α, Peroxisome proliferator-activated receptor alpha, STAC: SIRT1-activating compound; TG, triglycerides; T2DM: type 2 diabetes mellitus.
